# A review of stem cell therapy: An emerging treatment for dementia in Alzheimer's and Parkinson's disease

**DOI:** 10.1002/brb3.2740

**Published:** 2022-08-15

**Authors:** Aishwarya Umesh Pradhan, Olivier Uwishema, Helen Onyeaka, Irem Adanur, Burhan Dost

**Affiliations:** ^1^ Oli Health Magazine Organization Research and Education Kigali Rwanda; ^2^ Department of Research and Project Clinton Global Initiative University New York New York; ^3^ Faculty of Medicine Karadeniz Technical University Trabzon Turkey; ^4^ School of Chemical Engineering University of Birmingham Edgbaston Birmingham UK; ^5^ Department of Anesthesiology School of Medicine Ondokuz Mayis University Kurupelit Samsun Turkey

**Keywords:** Alzheimer's disease, dementia, Lewy body dementia, Parkinson's disease, stem cells

## Abstract

**Aim:**

This article aims to study the benefits and disadvantages of stem cell therapy, especially for patients who have dementia.

**Methods:**

The databases PubMed, Google Scholar, and the National Library of Medicine were searched for literature. All papers on Alzheimer's disease, Lewy body dementia, Parkinson's disease, stem cell therapy, and its effect on dementia treatment were considered.

**Results:**

Stem cell treatment has demonstrated promising outcomes in animal studies by positively modifying the degenerative alterations in dementia. However, it is not without drawbacks, such as ethical concerns while using embryonic stem cells and the danger of developing cancer if the cells undergo uncontrolled differentiation.

**Conclusion:**

Although stem cell therapy has its risks, it has the potential to be a viable therapeutic option for patients with dementia if developed appropriately. Hence, more research and clinical trials are needed to establish its efficacy in this context.

## 1 INTRODUCTION

Dementia may be marked by disorientation, behavioral changes, and cognitive disturbances and is currently said to have affected around 50 million people around the globe, and it is predicted that by 2050, the number will increase to 132 million (Biehl & Russell, 2009). Dementia adversely affects the social and occupational life of patients (Duncan & Valenzuela, 2017). Alzheimer's disease (AD) and Parkinson's disease (PD) are two examples of neurological conditions that present with dementia. It is estimated that 5.8 million Americans have Alzheimer's disease, the most common cause of dementia (Duncan & Valenzuela, 2017), while 1% of adults over 60 years of age are reported to have Parkinson's disease (Desplats et al., 2009).

Alzheimer's disease is characterized by atrophy and gliosis of hippocampus and medial temporal lobe early in the disease course. Eventually, the cortex of the frontal and parietal lobe, followed by the occipital lobe, is affected as well. Histological features include amyloid plaques, extracellular eosinophilic deposits of Aβ peptide containing amyloid and neurofibrillary tangles formed by abnormally modified microtubule‐associated tau protein aggregates within the neural tissue (Chagastelles & Nardi, 2011). Mutations in presenilin‐1 (PSEN1), presenilin‐2 (PSEN2) and rarely amyloid precursor protein (APP) are found in less than 5% of patients with AD (Biehl & Russell, 2009). Moreover, impairment of function and cognition in AD also correlates with the cerebral loss of neurons and synapses (Lees & Smith, 1983). The pathological changes in PD involve a reduction in the number of dopaminergic neurons in substantia nigra (Desplats et al., 2009) and α‐synuclein containing Lewy bodies (Mazini et al., 2019, Guo & Lee, 2014).

Current pharmacological therapy available for conditions presenting with dementia, such as Alzheimer's disease and Parkinson's disease, is primarily symptomatic with minimal side effects and has undergone outstanding progress. However, there have been limited advances in designing treatments to avoid neuronal loss, which is a known degenerative alteration in these diseases (Lee et al., 2010).

The capacity for regeneration of stem cells and their efficacy and safety when applied in therapy have already been reported by several studies in the past (Lee et al., 2010), making it a potential option for targeting neurodegeneration in AD and PD.

This article will discuss stem cell‐based therapy and its effect on preventing or repairing the pathological changes that occur in AD and PD and its potential role as a treatment modality in these conditions.

## 2 METHODOLOGY

### 2.1 Literature search

The data for this narrative review article was searched using the following databases: PubMed, Google Scholar, Scopus, and National Library of Medicine. The terms included in the search included stem cells, dementia, Alzheimer's disease, Lewy body dementia, Parkinson's disease, and stem cell therapy. Literature search and data collection were conducted between March 24 and April 5, 2022.

### 2.2 Inclusion criteria

The studies included according to the inclusion criteria were (i) articles discussing stem cell therapy in Alzheimer's disease and Parkinson's disease, (ii) the epidemiology of Alzheimer's disease and Parkinson's disease, (iii) pathology of Alzheimer's disease and Parkinson's disease, and (iv) articles published in English.

### 2.3 Exclusion criteria

Commentaries, nonclinical investigations and editorials and articles published in languages other than English were not included.

### 2.4 Study selection

The inclusion and exclusion criteria were independently implemented by three authors (AUP, OU, and HO).

### 2.5 Difference of opinion

There was no difference of opinion among the authors.

## 3 CLASSES OF STEM CELLS

According to their origin, stem cells are categorized as either adult (somatic) cells or embryonic stem cells (Arvanitakis et al., 2019). Embryonic stem cells are procured from blastocysts and can differentiate into any cell type. Adult stem cells, often referred to as somatic stem cells, are derived from adult tissues, most often bone marrow, and have a limited potential for regeneration (Uwishema et al., 2022). Controversy about these terms is that adult stem cells, even if completely differentiated, can be converted to their embryonic form using recently found research techniques. In addition, adult stem cells can also be present in fetal tissue, the placenta, and umbilical cord blood. As a result, a more accurate categorization is created based on stem cell potency (Arvanitakis et al., 2019).

Based on their potency, stem cells are grouped into pluripotent and multipotent. Pluripotent stem cells may develop into any form of cell. These cells are not present in the embryo for long periods as they develop and generate multipotent stem cells. The latter is involved in developing several specialized tissues throughout the body (Arvanitakis et al., 2019).

## 4 THERAPEUTIC APPLICATION OF STEM CELLS

Because of their potential to multiply and give rise to multiple cell types, stem cells are an intriguing prospect for repairing damaged tissues. Stem cell therapy is increasingly being researched as a treatment option in neurocognitive disorders presenting with dementia, such as AD and PD.

Majority of the available research at present focuses on mesenchymal stem cells (MSC), which are pluripotent and aid in neurogenesis and angiogenesis. They also prevent the loss of neurons by exerting anti‐apoptotic effects. This is brought about by releasing growth factors, neurotrophins, and cytokines. Thus, they aid in remyelination and regeneration. Additionally, they also interact with several immune cells, thus giving rise to anti‐inflammatory effects (Lee et al., 2010).

There are different types of mesenchymal stem cells based on the tissue that they are sourced. Several clinical trials studied and compared bone marrow‐derived MSC (BMMSC), adipose‐derived MSC (ADMSC), umbilical cord‐derived MSC (UC‐MSC), and placenta‐derived MSC (PD‐MSC) (Lee et al., 2010, Guillot‐Sestier et al., 2015, Mezey et al., 2000). BMMSC and ADMSC were the most dependable and widely utilized among them (Lee et al., 2010, Guillot‐Sestier et al., 2015).

### 4.1 Stem cell therapy in Alzheimer's disease

Processes involved in the pathogenesis of AD include proliferation, apoptosis, angiogenesis, inflammation, immunomodulation and so on. It is proposed that stem cell transplantation may alter these processes, thus repairing the neurological dysfunction and bringing about improvement in neurobehavioral function (Lees & Smith, 1983).

#### 4.1.1 Removal of Aβ plaques

Research on animal models of Alzheimer's disease has proposed that Aβ‐peptides may trigger apoptosis of neurons via oxidative stress (Goldberg et al., 2015, Oh et al., 2016). In addition, some studies suggest that transplanted stem cells may stimulate enzymes such as neprilysin‐degrading enzyme, insulin‐degrading enzyme, and endothelin converting enzymes, which have Aβ‐degrading characteristics. Stem cells thus contribute to the reduction of hippocampal Aβ plaques (Harach et al., 2017, Oh et al., 2016).

#### 4.1.2 Immunomodulatory actions

BMMSCs were able to speed up the activation of microglia, which participate in the removal of Aβ deposits in the brains of AD patients (Okazaki et al., 2008). This finding was suggestive of the fact that BMMSCs are also capable of demonstrating immunomodulatory actions (Okazaki et al., 2008, Oh et al., 2016).

#### 4.1.3 Anti‐inflammatory effects

Furthermore, IL‐10 signaling, although having anti‐inflammatory actions, was found to be extremely high in AD patients. The levels of IL‐10 were found to decrease after human stem cell transplantation (hMSC), which led to the hypothesis that blocking IL‐10 could be therapeutically relevant in AD. Likewise, TNFα, which is involved in chronic inflammatory processes and cancer, was also decreased following hMSC transplantation (Safar et al., 2016).

#### 4.1.4 Inhibitory effect on apoptosis

Cognitive dysfunction in AD can be correlated with neuronal apoptosis. According to some findings, BMMSCs reduce caspase‐3 expression (Chen et al., 2003), boost cell survival signals (Wang et al., 2010), and produce vascular endothelial growth factor (VEGF), brain‐derived neurotrophic factors (BDNF), nerve growth factor (NGF), and fibroblast growth factor 2 (FGF‐2) (Elahi & Miller, 2017), which possibly aids BMMSCs in diminishing Aβ peptide deposition (Lees & Smith, 1983).

#### 4.1.5 Neurogenesis

Intravenously infused BMMSCs were able to migrate and reach the brain at the site of injury (Oh et al., 2016), where they differentiated into neuron‐like cells and partially expressed choline acetyltransferase (ChAT) (Lees & Smith, 1983). The expression of ChAT is significant as it could be a potential mechanism of neurogenesis following transplantation of BMMSCs (Yang et al., 2004).

Moreover, expression of growth factor, chemokine, and extracellular matrix receptors on MSC surface could lead to production and upregulation of factors such as NGF, FGF‐2, insulin like growth factor 1 (IGF‐1), and BDNF, in turn facilitating endogenous regeneration and recovery of neurological function (Oh et al., 2016).

#### 4.1.6 Angiogenesis

The presence of Aβ reduces the amount of soluble VEGF in the brain by binding to it and forming aggregates which ultimately lead to the loss of angiogenesis (Uwishema et al., 2022). hMSCs increased the expression of VEGF and vascular endothelial growth factor receptor‐2 (VEGFR2) in ischemic areas of the brain (Qin et al., 2020, Nagaya et al., 2004), and increased the formation of peripheral vascular layers by differentiating into mural cells (Lee et al., 2009).

Secretion of epidermal growth factor (EGF), neurotrophin‐3(NT‐3), hepatocyte growth factor (HGF), VEGF, FGF‐2, and BDNF by mesenchymal stem cells is thought to have a neuroprotective effect in neurodegenerative disorders (Vanhelleputte et al., 2003).

### 4.2 Stem cell therapy in Parkinson's disease

The presence of α‐synuclein is associated with cognitive impairment in Parkinson's disease dementia (PDD) (Kern et al., 2006). Although some studies have shown that transplantation of dopaminergic precursors caused improvement in motor symptoms of PD (Chen et al., 2004), studies evaluating the potential of stem cell transplantation in treating cognitive dysfunction in PDD are very few (Kern et al., 2006).

α‐synuclein aggregates are known to be toxic to cells, leading to neuronal deaths in many α‐synucleinopathies (Hirschi et al., 2003). Neuronal cells release α‐synuclein aggregates by the process of exocytosis (Hirschi et al., 2003), which are then taken up via endocytosis by neurons and glial cells (Van den Bos et al., 2022, Salem et al., 2014, Mo et al., 2012). Furthermore, it is suggested that the interaction between α‐synuclein and N‐methyl‐D‐aspartate (NMDA) receptors could facilitate clathrin‐mediated endocytosis of NMDA receptors (Gomperts, 2016).

Clathrin and early endosome antigen 1 (EEA1) expression was found to be increased in cells treated with α‐synuclein. This expression, however, was significantly decreased when cells were cocultured with MSCs. The internalization of α‐synuclein via clathrin‐mediated endocytosis was also inhibited by transplanted mesenchymal stem cells. This was observed to occur by altering the interaction between α‐synuclein and NMDA receptors, thus reducing α‐synuclein transmission and cell death induced by it (Hirschi et al., 2003).

## 5 OBSTACLES ENCOUNTERED IN STEM CELL THERAPY

Even though stem cell therapy has its advantages, establishing stem cell lines is a complicated process.

One such difficulty is faced while maintaining a suitable environment in which embryonic stem cells can remain undifferentiated. After development, embryonic stem cell lines are generally cultured indefinitely or are frozen and thawed. Another hurdle is the ethical concern arising with the use of blastocysts as the consideration that they constitute a human being is not entirely dependent on scientific studies (Uwishema et al., 2022).

Pluripotent stem cells are sourced from a donor, thus posing a risk of immune‐mediated rejection of the graft used. This warrants the long‐term use of immunosuppressant drugs. Teratoma is also a significant concern of pluripotent cells, which emerges as a result of their potential to differentiate into a wide range of specialized cells. Owing to the risk of developing teratomas, animal studies that evaluated the therapeutic potential of pluripotent stem cells failed to yield positive results. However, the risk of this complication can be minimized by reducing the proliferative potential of these stem cells. In contrast, multipotent stem cells are extracted from self‐tissue (autologous), avoiding the risk of rejection. Because of their limited proliferative power and propensity to specialize in blood cell precursors, multipotent stem cells have been investigated for the treatment of leukemia, lymphoma, and myeloma since the 1960s (Arvanitakis et al., 2019).

Bone marrow aspiration without local anesthesia is accompanied by pain of moderate‐intensity (Cheng et al., 2011), which may prove to be a hurdle in acquiring BMMSCs. This can be prevented or alleviated by prescribing tramadol before the procedure.

## 6 CONCLUSION

Dementia is already a worldwide issue causing considerable impairment in the daily functioning of patients. Unfortunately, the numbers are expected to rise threefold in the coming years.

With treatments available to manage the symptoms of Alzheimer's disease and Parkinson's disease, the two most important etiologies of dementia, it is critical to investigate options for avoiding or minimizing the pathological events that happen in the nervous system with these disorders. Studies suggest that stem cell therapy alters these changes due to their regenerative and proliferative properties.

Although this therapy has its disadvantages, such as the risk of rejection and developing cancer, more research and clinical trials should be conducted in this domain to discover a technique that would exploit the desirable traits of stem cell therapy while trying to lessen the risks associated with it.

## AUTHOR CONTRIBUTIONS

Olivier Uwishema: Conceptualization, project administration, writing‐review, and designing. Olivier Uwishema: Reviewed and edited the first draft. Aishwarya Umesh Pradhan: Reviewed and edited the second draft. Helen Onyeaka: Reviewed and edited the final draft. All authors: Data collection and assembly, manuscript writing, and final approval of manuscript.

## CONFLICT OF INTEREST

No conflicts of interest declared.

### PEER REVIEW

The peer review history for this article is available at https://publons.com/publon/10.1002/brb3.2740.
